# Methylmercury Exposure and Adverse Cardiovascular Effects in Faroese Whaling Men

**DOI:** 10.1289/ehp.11608

**Published:** 2008-10-16

**Authors:** Anna L. Choi, Pal Weihe, Esben Budtz-Jørgensen, Poul J. Jørgensen, Jukka T. Salonen, Tomi-Pekka Tuomainen, Katsuyuki Murata, Hans Petur Nielsen, Maria Skaalum Petersen, Jórun Askham, Philippe Grandjean

**Affiliations:** 1 Department of Environmental Health, Harvard School of Public Health, Boston, Massachusetts, USA; 2 Faroese Hospital System, Tórshavn, Faroe Islands; 3 Institute of Public Health, University of Copenhagen, Copenhagen, Denmark; 4 Department of Clinical Biochemistry, Pharmacology, and Genetics, Odense University Hospital, Odense, Denmark; 5 Oy Jurilab Ltd., Kuopio, Finland; 6 Research Institute of Public Health, School of Public Health and Clinical Nutrition, University of Kuopio, Kuopio, Finland; 7 Department of Environmental Health Sciences, Akita University School of Medicine, Akita, Japan; 8 Institute of Public Health, University of Southern Denmark, Odense, Denmark

**Keywords:** cardiovascular physiology, evoked potentials, food contamination, methylmercury, neurotoxicity, pilot whale, seafood

## Abstract

**Background:**

Methylmercury (MeHg), a worldwide contaminant found in fish and seafood, has been linked to an increased risk of cardiovascular mortality.

**Objective:**

We examined 42 Faroese whaling men (30–70 years of age) to assess possible adverse effects within a wide range of MeHg exposures from consumption of pilot whale meat.

**Methods:**

We assessed exposure levels from mercury analysis of toenails and whole blood (obtained at the time of clinical examination), and a hair sample collected 7 years previously. Outcome measures included heart rate variability (HRV), blood pressure (BP), common carotid intima-media thickness (IMT), and brainstem auditory evoked potentials (BAEP). We carried out multiple regression and structural equation model (SEM) analyses to determine the confounder-adjusted effect of mercury exposure. Taking into account correlations among related measures, we categorized exposure and outcomes in groups to derive latent exposure and response variables in SEMs. We used multiple regression analysis to compare the predictive validity of individual exposure biomarkers and the latent exposure variable on individual and latent outcomes.

**Results:**

The toenail mercury concentrations varied widely and had a geometric mean of 2.0 μg/g; hair concentrations averaged about 3-fold higher. Mercury exposure was significantly associated with increased BP and IMT. This effect was reflected by SEMs, but mercury in toenails tended to be the best effect predictor.

**Conclusions:**

The results support the notion that increased MeHg exposure promotes the development of cardiovascular disease.

Methylmercury (MeHg), a worldwide contaminant found in fish and seafood, has been linked recently to adverse effects on the cardiovascular system. Increased risk of coronary heart disease was found with elevated hair-mercury concentrations in a cohort of Finnish men, whose increased MeHg exposures were largely from consumption of fish from contaminated lakes (Salonen et al. 1995). Association with accelerated progression of carotid atherosclerosis, as measured by common carotid intima-media thickness (IMT), was later found in the same Finnish population (Salonen et al. 2000). MeHg exposures, as reflected by toenail mercury concentrations, were also associated with a higher risk of myocardial infarction in a European multicenter study (Guallar et al. 2002), and a similar, although insignificant association was found in nondentist health professionals in the United States (Yoshizawa et al. 2002). MeHg-associated increase in blood pressures (BPs) was observed in a prospective cohort study of Faroese children at 7 years of age, and a decrease in heart rate variability (HRV), a marker of cardiac autonomic control, occurred both at 7 and 14 years of age (Grandjean et al. 2004; Sørensen et al. 1999). These observations suggest that MeHg may be an important risk factor for cardiovascular disease, although results have not been consistent (Hallgren et al. 2001). The pathogenesis of MeHg-associated cardiovascular abnormalities is unclear, but it may involve neurotoxic effects. Thus, associations between HRV and brainstem auditory evoked potential (BAEP) latencies in MeHg-exposed adolescents suggest a link between cardiovascular autonomic tone and brainstem neurotoxicity (Grandjean et al. 2004; Murata et al. 2004).

Although MeHg exposure originates from contaminated seafood and freshwater fish, a high fish intake is also thought to provide protective effects against coronary heart disease (Burr et al. 1989; Mozaffarian and Rimm 2006; Oomen et al. 2000). Adverse effects of MeHg from fish and seafood (levels of which vary widely across species; generally fish that are high in the food chain have higher MeHg concentrations) could therefore counteract the benefits from nutrient contents, such as omega-3 fatty acids and selenium (National Research Council 2000; Salonen et al. 1995). Thus, the somewhat equivocal results of epidemiologic studies relating fish consumption and cardiovascular events (Marckmann and Grønbaek 1999) could be due to interference from MeHg toxicity. Evidence from the few studies that assessed both beneficial and adverse cardiovascular effects (Salonen et al. 1995; Yoshizawa et al. 2002) support this notion.

To explore MeHg-associated effects on cardiovascular functions (CVFs), we carried out a clinical study of male members of the whaling society in the Faroe Islands. This Nordic fishing community exhibits a wide range of MeHg exposure that primarily originates from consumption of pilot whale meat; other types of seafood contain lower MeHg concentrations (Grandjean et al. 1992). Members of the Faroese whaling society organize and participate in the whale drives and receive a larger share of the whale meat and blubber, although the availability varies across islands and over time. We used mercury analyses of hair, blood, and toenail samples to ascertain the individual exposure levels and also included the result of a hair analysis conducted 7 years earlier. We then used a structural equation model (SEM) analysis to assess the association between the combination of MeHg exposure biomarkers on individual and groups of outcome variables.

## Materials and Methods

### Study population

The study population originally consisted of 70 male members of the Faroese whaling society who had volunteered for an exposure examination (hair was obtained for mercury analysis) in 1998. The hair-mercury concentrations varied between 2.6 and 54.1 μg/g (mean, 15.9 μg/g) and thus documented an increased level of exposure from frequent consumption of pilot whale meat. We invited the subjects for a clinical examination in 2005. Sixteen subjects did not participate; reasons included death (4), emigration or long-term travel (6), refusal (4), lost to follow-up (1), or exclusion (1 female). Of the 54 male participants, 42 were between 30 and 70 years of age. The Faroese Ethical Review Committee and the Institutional Review Board at the Harvard School of Public Health approved the study protocol, and we obtained written informed consent from all participants.

As part of a general health examination, we recorded relevant covariates based on prior knowledge of potential influence on the cardiovascular and neurophysiologic outcome variables (Araki and Murata 1993; Salonen et al. 2000). Characteristics of the subjects included age, smoking history (with three categories: past smoker, present smoker, and nonsmoker), and body mass index (BMI; calculated as the body weight in kilograms per height in meters, squared. Weekly alcohol consumption was categorized as *a*) none, *b*) ≤21 drinks, and *c* ) > 21 drinks according to the World Health Organization (WHO) guidelines (Goldberg et al. 2000), where up to 21 drinks per week is considered as low level of risk for men. To adjust for the nutrient benefits by fish intake, we included in the models the number of fish dinners consumed per week in the last year. This variable was very similar to the weekly fish intake as an adult but with more complete data. In addition, we measured fasting serum concentrations of cholesterol, cholesterol fractions, and triglycerides.

### Measurements of exposure

To ascertain long-term exposures to MeHg from food, we collected toenails, scalp hair, and whole blood for mercury analysis. The toenail clippings were collected from all toes and washed in a sonicator with deionized water before mineralization and analysis. The normal growth rate for toenails is 3–12 months (Karagas et al. 2000). Because toenails incorporate mercury during formation but are of different lengths, nail clippings taken from all toes at the same time reflect the incorporation of mercury that has occurred over approximately 1 year (Yoshizawa et al. 2002). In addition to the previously analyzed hair sample, an additional hair sample was cut from the occipital area and the proximal 2 cm were used for mercury analysis. Nail clippings and hair samples were stored dry at room temperature in a marked paper envelope until analysis. Whole blood was collected in a Venoject vial certified to be free of mercury. We used flow-injection cold-vapor atomic absorption spectrometry after digestion of the samples in a microwave oven to ascertain mercury levels (Dalgård et al. 1994; Grandjean et al. 1992, 1995). The relative analytical imprecision for this analysis was estimated to be 6.1%, 4.1%, and 7.4% at mercury concentrations of 0.35, 4.6, and 11.7 μg/g, respectively. We ensured the accuracy by measuring certified reference material BCR 397 (IRMM, Geel, Belgium), where our result was 11.74 ± 0.87 μg/g, compared with the certified value of 12.3 ± 0.5 μg/g. (Mercury concentrations reported in units of micrograms may be converted to nanomoles by multiplying by 5.0.)

Because pilot whale blubber contains elevated concentrations of polychlorinated biphenyls (PCBs), we also measured the serum concentrations of major PCB congeners. The total PCB concentration was calculated as twice the sum of congeners 138, 153, and 180 and expressed on a lipid-weight basis (Heilmann et al. 2006).

### Outcome measurements

#### CVF assessment

At a general health examination, the systolic and diastolic BPs were measured under standardized conditions with the subject relaxing in a chair. A cuff that covered between one-half and two-thirds of the upper arm was applied on the left arm, and the pressures were read in millimeters of mercury on a sphygmomanometer. Body weight in kilograms was measured on an electronic scale to the nearest 100 g. Standing height was measured with a stadiometer to the nearest millimeter. All outcome parameter assessments were performed blind with regard to MeHg exposures.

The R-R intervals (sampling time, 1 msec) during 5 min were measured using the Heart Rate Monitor S810 and Polar S-series Precision Toolkit (Polar, Kempele, Finland), with the subject resting in a supine position. Using the electrocardiogram (ECG) R-R Interval Analyzer (Grandjean et al. 2004; Murata et al. 2006), 100 consecutive R-R intervals with the minimal SD were automatically extracted for calculation of the average heart rate and its relative SD. The coefficient of variation (CVRR) is the ratio of the SD of the R-R intervals to the average value, and was also expressed as a percentage, CVRR% (Grandjean et al. 2004). We applied autoregressive spectral analysis to partition the HRV into independent components as indicators for parasympathetic and sympathetic activities (Akselrod 1995; Murata et al. 1992). The results of the spectral analysis were expressed in low-frequency (0.01–0.15 Hz) and high-frequency (0.15–0.40 Hz) components. We defined the coefficient of variation for the low-frequency band (C-CVLF) and high-frequency band (C-CVHF) as the ratio of the square root of the power spectral density of each component to the R-R mean. The C-CVLF and C-CVHF are thought to reflect mainly sympathetic and parasympathetic activities, respectively (Grandjean et al. 2004; Murata et al. 2006).

The IMT of the carotid arteries was assessed as an indicator of carotid atherosclerosis (Salonen et al. 2000). The subjects, in a supine position, were examined by high-resolution B-mode ultrasonography of the right and left common carotid arteries (CCA) in a 1.0–1.5 cm section at the distal end of the CCA, which was conducted with the HP Sonos 5500 ultrasound system with a trapezoidal linear array 3–11 MHz transducer. The IMT, assessed by computerized analysis of the ultrasound images with a semiautomated border detection software, PROSOUND (University of Southern California, Los Angeles, CA, USA) (Selzer et al. 1994), is the mean distance between the intima-lumen and media-adventitia interfaces measured at approximately 100 points per 1-cm section in both the right and left CCAs (Salonen et al. 2000). We used three measures of IMT for the present study: *a*) mean IMT (the mean of all IMT estimates from the left and right CCAs, considered an overall measure of the atherosclerotic process), *b*) mean maximal IMT (the mean of the points of maximal thickness from the right and left CCAs), and *c*) mean minimal IMT (the mean of the points of minimal thickness from the right and left CCAs).

#### Neurophysiologic assessment

BAEP latencies have been linked with MeHg-related changes in HRV, possibly reflecting MeHg neurotoxicity exerted in brainstem nuclei (Grandjean et al. 2004; Murata et al. 2004). BAEP assessments were determined in comfortably resting subjects using a four-channel electromyography (Medelec Sapphire-4ME, Surrey, UK). Similar equipment was used previously with other cohorts (Grandjean et al. 1997; Murata et al. 2004). Click signals at an intensity of 65 dB hearing level (0.1-msec impulses of alternating polarity) were presented to the right ear through shielded ear phones at rates of 20 Hz/sec and 40 Hz/sec independently (sampling time, 0.01 msec). The left ear was masked with white noise at an intensity of 45 dB HL. BAEP latencies were recorded by using three standard electroencephalogram electrodes placed on the vertex, the right mastoid ipsilateral to the stimulation, and the left mastoid (ground). The responses were averaged 2,048 times after amplification and filtration (bandpass, 200–2,000 Hz), with replication for each rate to calculate average peak latencies. Peaks I, III, and V are thought to reflect the volume-conducted electric activity from the acoustic nerve, pons (superior olivary nucleus), and midbrain (inferior colliculi), respectively (Murata et al. 2004).

### Statistical analyses

The distributions of the mercury concentrations were skewed, and were therefore log (base 10) transformed. This transformation is required by the SEM analysis to obtain approximately linear relationships with homogeneous scatter between the exposure biomarkers. Similarly, most of the outcome variables were log transformed except for the BP and heart rate variables, which approximated a Gaussian distribution. We calculated geometric means for the transformed variables and arithmetic means for BPs.

Because of the availability of several exposure parameters and several outcome variables, standard regression analysis with confounder adjustment was complemented by SEM to assess the association between an integrated set of biomarkers of mercury exposure on the one hand, and sets of cardiovascular and neurophysiologic outcomes on the other. SEMs have only recently been introduced in environmental health research, but detailed instructions are available (Bollen 1989; Budtz-Jørgensen et al. 2002). The observed variables are considered to be manifestations of a smaller number of latent variables (which are not available for direct observation but can be estimated from the observed variables). In the case of mercury exposure, we have


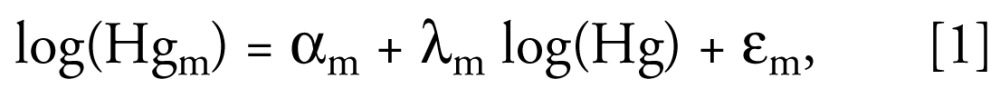


where Hg_m_ is a marker of mercury exposure, and Hg is the “true” (latent) exposure. The log-transformed mercury biomarker depends linearly on the latent (log-transformed) mercury exposure and a measurement random error (ɛ_m_). As a starting point, error terms in different markers are assumed to be independent. We fixed the factor loading λ_m_ at 1 for toe-nail mercury, so that the latent exposure was expressed on the scale of the mercury concentration in nails. Hence, a one-unit increase in log(Hg) will on average lead to a one-unit increase in log nail mercury. α_m_ is the intercept. We fixed this parameter to zero for the log nail mercury concentration, indicating that the latent exposure also has the same zero point as the log nail concentration. The biomarkers can be compared in terms of both their imprecision and their estimated correlations with the true exposure (Budtz-Jørgensen et al. 2003). The exposure biomarker with the smallest imprecision and the highest correlation to the latent MeHg exposure is the best indicator.

The relationship between the latent variables is then considered after adjustment for the effects of covariates. SEM analysis allows the integration of different measures of exposure to generate a latent exposure variable that likely provides a closer approximation of the true exposure than individual exposure measures considered alone. In addition to addressing the issue of exposure imprecision, the SEM model addresses multiple testing and missing data issues that may not be adequately considered by standard regression analyses (Budtz-Jørgensen et al. 2002). We included information from incomplete cases using the maximum likelihood approach (Little and Rubin 2002). However, such subjects were excluded in the standard regression analysis. For this reason, the standard regression models may be inefficient and even biased if information is not missing completely at random. Additional bias is also possible, because imprecision of the exposure assessment is not included.

Likewise, the outcome variables were grouped into a small number of latent variables assumed to reflect broad groupings of physiologic functions. The neurophysiologic variables were collected into a joint BAEP latent variable. The cardiovascular outcomes were grouped into heart rate variability, as HRV (CVRR%, C-CVLF, and C-CVHF), CVF (systolic and diastolic BP, and heart rate), and carotid atherosclerosis (mean, maximal, and minimal IMT).

We constructed a model linking a mercury latent variable with the three cardiovascular and the BAEP groupings considered as independent latent functions. Potential confounders were included in the SEM as covariates. PCB was considered as a potential additional confounder in separate analyses. Adjustment or local dependence between outcomes in each grouping was considered if the correlation between the outcomes could not be fully explained by the underlying cardiovascular or neurophysiologic functions by using the likelihood ratio test (Bollen 1989; Budtz-Jørgensen et al. 2002).

To compare the SEM approach with linear regression analyses, we used separate regression models to assess associations between latent and individual mercury exposure parameters and outcomes, after confounder adjustment. Because the outcomes were not of the same magnitude and transformations had been used, and because the loading factors for the two exposure variables (mercury in whole-blood and hair samples taken 7 years earlier) were lower than that of toenail mercury, regression coefficients were standardized. Thus, we estimated the change (in percentages of the SD of the unadjusted outcome parameter) in the outcome associated with 1 SD increase in the MeHg exposure variable. Two-sided tests for statistical significance at *p* < 0.05 were performed. Descriptive analyses and linear regression models were carried out in SAS (version 9.1; SAS Institute Inc., Cary, NC, USA). SEMs were developed in Mplus (version 3.1; Muthen & Muthen, Los Angeles, CA, USA).

## Results

Table 1 shows the characteristics of the 42 male members of the Faroese whaling society examined in this study. Among the 41 men with detailed whale consumption data, 26 (63%) consumed three or more whale meals per month. We included the binary (yes/no) weekly alcohol consumption variable in the models for model robustness because the three-categories alcohol variable had no significant associations with the outcomes and the effects of mercury changed only marginally after its inclusion as a confounder in the models. Serum concentrations of cholesterol and triglycerides were considered normal for the age groups examined and were only weakly correlated with indicators of mercury exposure (correlations ranged from 0.07 to 0.16, *p* > 0.3).

Table 2 shows the geometric mean and interquartile range of the mercury concentrations. All exposure biomarkers showed relatively wide ranges, where the highest mercury concentration was 50-fold higher than the lowest in toenails and hair. Mercury levels in hair samples taken 7 years earlier were higher than the levels of the current hair samples. Among the four exposure biomarkers, mercury in toenails showed the closest correlation with hair mercury levels. The average ratio of mercury concentrations in hair to those in toenails was approximately 3. The PCB concentrations were comparatively high and were positively associated with the mercury concentrations (correlation coefficients between 0.47 and 0.54, *p* < 0.002).

The mean IMT was highly correlated with the maximal and minimal measures (Table 3). We anticipated the results because all three outcomes measured the distance from the right and left CCAs but with different summary estimates. Likewise, correlations between CVRR%, C-CVLF, and C-CVHF were high as components of HRV (Table 3). The systolic and diastolic BPs correlated well (*r* = 0.79) and were weakly and negatively correlated with CVRR. The latencies of peaks III and V at 20 Hz and 40 Hz were highly correlated (Table 3), and the mean latencies were similar to those of a study of Faroese children at 14 years of age (Murata et al. 2004).

Mercury concentrations in current hair and whole-blood samples were highly correlated (*r* = 0.94, *p* < 0.001). To obtain an SEM consisting of exposure variables with independent error terms, we included the results only for toenails, blood, and hair from 7 years previously. This model showed that toenail measurement had the smallest error component. Thus, the toenail mercury biomarker had the highest correlation with the estimated latent exposure (0.98, compared with 0.83 for blood and 0.46 for hair from 7 years before), thereby suggesting that it is the best indicator of long-term MeHg exposure in this population. When combined with the outcome variables, the results again showed that, among the three mercury biomarkers, toenail mercury measurement was the best indicator, with the smallest imprecision and the highest correlation to the latent MeHg exposure (Table 4).

**Figure 1:**
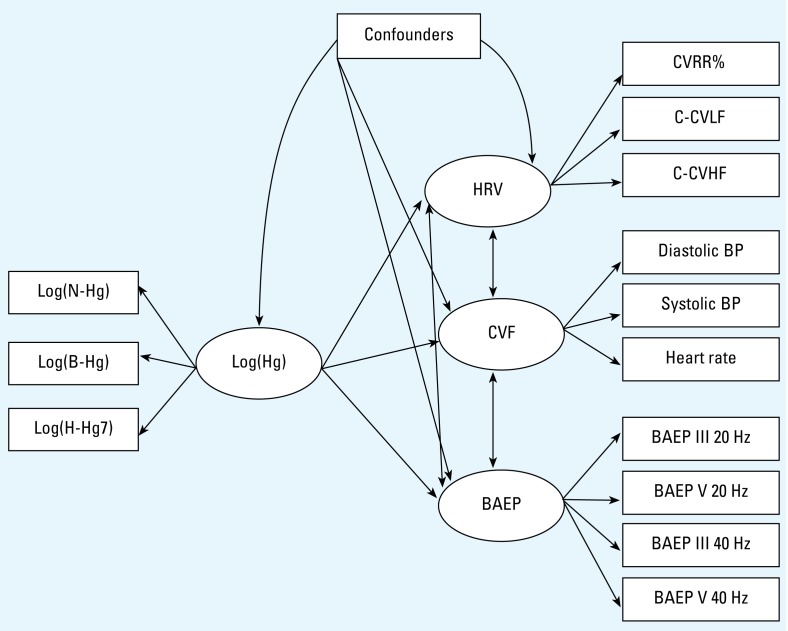
Path diagram for an SEM linking mercury exposure to adverse effects, with adjustment for confounders. The estimated true exposure (Hg) is modeled as a latent parameter based on mercury concentrations in toenail (N-Hg), blood (B-Hg), and hair sample taken 7 years ago (H-Hg7). Three latent effect parameters, HRV, CVF, and latencies of BAEP, are expressed by a series of heart variability, cardiovascular, and BAEP (peaks III and V at both 20 and 40 Hz) test results, respectively. HRV, CVF, and BAEP may be correlated (indicated by double-headed arrows), and potential confounders are allowed to be associated with both the latent exposure variables and the latent effect variables.

An SEM was constructed to determine the overall effect of mercury exposure on the groups of outcome variables with adjustment for confounders (Figure 1). PCB exposure showed no statistically significant associations with the outcomes, and inclusion of PCB as a confounder in the models changed the effects of mercury only marginally. The results are therefore reported without PCB adjustment. High correlations among related measures within this small sample size limited the number of outcome variables that could be included in latent variables. We included all variables shown in Figure 1 in the final model (Table 4). Among the HRV indicators, CVRR% was the best indicator of the combined outcomes, with the smallest imprecision and the highest correlation to the latent HRV variable. Likewise, peak V at 20 Hz has slightly better correlation with the latent BAEP latent variable. For CVF, the diastolic BP and heart rate were the better indicators.

For comparison, results are reported both for major individual effect variables and for the latent variables (Table 5). We also performed separate models for the latent exposure variable with each of the outcome groupings and obtained similar results. Among the three mercury measures, the coefficients from the regression models revealed some differences. For the CVRR% outcome, for example, the coefficient for the blood concentration was in the direction opposite of expectation, but was close to zero for other exposure biomarkers and the latent exposure variable. Similar results were obtained with the other two HRV measures. In contrast, for both CVF and IMT, tendencies are similar for the three individual exposure parameters and the latent exposure variable, thus supporting a causal linkage. The confidence intervals for the latent exposure interval also take into account the temporal variability of the exposures, and the wider confidence intervals are reflected by increased upper confidence limits. Because standard regression analysis fails to take into account measurement error in exposure variables, the confidence intervals may be too narrow.

## Discussion

In this article, we present results on long-term MeHg effects on cardiovascular and neurophysiologic functions in a small group of men exposed to dietary MeHg from whale meat as the main seafood source. Our findings suggest that MeHg may promote development of cardiovascular disease, as indicated by increases in BP and in IMT. BAEP latencies showed slight delays associated with MeHg exposure, with wide confidence limits, whereas effects on HRV were equivocal.

A major advantage of the present study is that we used several exposure biomarkers as indicators of long-term MeHg exposure. Past studies of adults have used mercury concentrations in hair (Salonen et al. 1995, 2000; Virtanen et al. 2005), blood (Hallgren et al. 2001), or toenails (Guallar et al. 2002; Yoshizawa et al. 2002). Because MeHg has an elimination half-life of about 1.5–2 months (National Research Council 2000), mercury incorporated in growing hair and toenails will reflect MeHg intakes during the months past (Garland et al. 1993; International Programme on Chemical Safety 1990). Although standard regression analysis assumes that each exposure biomarker is measured without imprecision, an SEM analysis uses integrated information from all available exposure biomarkers and takes into account the imprecisions originating from both laboratory measurement error and biological variation (Budtz-Jørgensen et al. 2002). If the model assumptions are appropriate, the latent variable is therefore likely to provide a better approximation to the true long-term MeHg exposure. Among the individual exposure parameters, toenail mercury appears to be the most precise biomarker, and the regression analyses showed effects of the toenail mercury on most of the outcomes that were similar to the effects of the latent MeHg exposure.

The SEMs showed clear effects on BP and on IMT. The association between MeHg exposure and increased BP is consistent with previous findings in a Faroese birth cohort (Sørensen et al. 1999). MeHg effects on carotid atherosclerosis, as measured by IMT, were indicated by significant associations with toenail and hair mercury concentrations. The results support and expand the result of the prospective study of Finnish men (Salonen et al. 2000).

As a marker of cardiac autonomic control (Jhun et al. 2003; Magari et al. 2002), HRV showed a surprising increase at higher blood-mercury concentrations that would reflect recent MeHg exposures. We found no such tendency for other MeHg exposure biomarkers or in the latent exposure variable. A lower CVRR has been previously linked to increased MeHg exposure in the Faroese birth cohort study (Sørensen et al. 1999). However, an effect in the opposite direction could conceivably be due to n-3 fatty acids from recent seafood intakes (Mozaffarian et al. 2008), thus possibly causing confounding with seafood-derived MeHg exposure.

BAEP latencies appeared to increase, although not significantly so, with increasing MeHg exposure. Prolonged BAEP latencies have been reported to have a direct association with MeHg exposure in animal and human studies (Chuu et al. 2001; Murata et al. 2004). The BAEP effects have been linked to exposure-related changes in HRV as a joint indication of MeHg neurotoxicity exerted in the brainstem nuclei (Grandjean et al. 2004; Murata et al. 2004). The present study is too small to explore this possible pathogenesis.

The Faroese whaling men had minimal social differences and exhibited a wide range of exposures, thus allowing for the examination of mercury effects on cardiovascular and neurophysiologic outcomes. Although the ages of the subjects differed considerably, even after exclusion of the very young and the very old, age was not significantly associated with MeHg exposure. We used HRV, IMT, and BP as outcome measures in our study because they are all well-established clinical parameters and are predictors of increased risk of adverse outcomes, and BAEP latencies are associated with HRV variables (Grandjean et al. 2004). The small sample size may have affected the model stability when attempting to include series of outcome data, such as the IMT and BPs (some of which were highly correlated) as latent variables.

The ratio of mercury concentrations in our current hair and blood sample was approximately 250:1, a finding that is consistent with the reported ratio for the human hair-mercury concentrations (in micrograms per gram) to whole-blood mercury concentrations (in micrograms per milli liter) under reasonably constant conditions of exposure [Budtz-Jørgensen et al. 2004; U.S. Environmental Protection Agency 2001; International Programme on Chemical Safety (IPCS) 1990].

The findings of the present study support previous studies at lower exposure levels. For example, the mean hair mercury concentrations were 1.92 μg/g for a Finnish cohort (Salonen et al. 1995), 2.99 μg/g and 0.92 μg/g for a Faroese birth cohort at 7 and 14 years of age, respectively (Debes et al. 2006, Grandjean et al. 1997), and 0.45 μg/g for nail mercury levels among a U.S. cohort of nondentist health professionals (Yoshizawa et al. 2002). In Finnish men, increased risk of cardiovascular death was seen at hair-mercury concentrations > 2 μg/g (Salonen et al. 1995). Using a conversion factor of 3 (Table 2), this exposure level corresponds to a toenail concentration of about 0.67 μg/g. Studies using toenail-mercury concentrations found an increased risk of heart disease at toenail mercury concentrations greater than 0.66 μg/g (Guallar et al. 2002) and 0.84 μg/g (Yoshizawa et al. 2002). For comparison, the reference dose used by the U.S. Environmental Protection Agency corresponds to 1 μg/g hair (National Research Council 2000), which would translate to about 0.33 μg/g in toenails. Although the toenail mercury appears to be a better risk indicator than other biomarkers, single mercury concentrations are imprecise measures of long-term exposure and will therefore result in an underestimated assessment of MeHg toxicity. An SEM provides a stronger and more reliable analysis by including all of the available measures of exposures and outcomes. In conclusion, at highly increased MeHg exposures, this study supports the notion that MeHg from seafood may promote the development of cardiovascular disease.

## Figures and Tables

**Table 1:** Characteristics of the 42 male members of the Faroese whaling society.

Characteristic	Values
Age [years (mean ± SD)]	54.8 ± 9.4
Weight [kg (mean ± SD)]	87.3 ± 10.7
Height [cm (mean ± SD)]	178.5 ± 6.4
BMI (mean ± SD)	27.4 ± 3.2
Smoking [none/past/current (%)]	41/33/26
Alcohol consumption per week [never/≤ 21 drinks/> 21 drinks (%)]	7/71/22
No. of fish dinners pr week [1/2/3–4 (%)]	24/44/32
Serum lipid concentrations [mmol/L (mean ± SD)]	
Total cholesterol	5.74 ± 0.98
High-density lipoprotein	1.47 ± 0.32
Low-density lipoprotein	3.62 ± 0.85
Triglycerides	1.49 ± 0.99

**Table 2:** Distribution of mercury and PCB concentrations in specimens used as exposure biomarkers among the 42 Faroese whaling men.

Exposure biomarker	Geometric mean	Interquartile range	Total range	Correlation with toenail mercury
Toenail (μg/g)	2.04	1.35–3.29	0.14–8.26	(1)
Blood (μg/L)	29.5	18.7–46.1	5.19–128.4	0.60
Hair (μg/g)			
Current	7.31	4.52–13.4	0.92–46.0	0.70
7 years before	13.9	9.80–21.9	4.80–43.7	0.56
Serum PCB^a^ (μg/g lipid)	10.8	6.6–16.8	1.13–42.4	0.54

a Calculated as twice the sum of PCB congeners 138, 153, and 180.

**Table 3:** Distribution of outcome variables among the 42 Faroese whaling men.

Outcome	Geometric mean	Interquartile range	Total range	Correlation^a^
IMT (mm)				
Mean	0.82	0.72–0.91	0.60–1.50	(1)
Maximum	1.09	0.98–1.17	0.78–2.35	0.92
Minimum	0.58	0.51–0.66	0.41–0.96	0.94
HRV				
CVRR%	2.99	2.14–4.34	1.26–8.59	(1)
C-CVLF	1.75	1.29–2.53	0.50–5.25	0.73
C-CVHF	1.30	0.90–1.77	0.27–3.91	0.79
CVF^b^				
Blood pressure (mmHg)				
Diastolic	85.0	80–90	65–105	(1)
Systolic	137.33	125–150	110–199	0.79
Heart rate (per second)	62.6	56–69	42–81	0.39
BAEP peak latency (msec)				
20 Hz				
I	1.82	1.65–1.92	1.57–2.55	0.67
III	4.15	3.95–4.32	3.65–4.96	0.85
V	6.07	5.85–6.37	5.57–6.74	(1)
40 Hz				
I	1.92	1.82–2.02	1.58–2.68	0.58
III	4.27	4.05–4.49	3.75–4.92	0.82
V	6.21	5.98–6.43	5.69–6.88	0.91

a Correlation of each variable with the referent variable in each group.

b For BPs and heart rate, the results are given as arithmetic means.

**Table 4:** Factor loadings and estimated correlation to each of the latent variables for mercury exposure and outcome groups in an SEM.^a^

Outcome	Factor loading (λ_m_)	Correlation to latent variable
Hg exposure		
Toenail (μg/g)	(1)	0.98
Blood (μg/L)	0.68	0.64
Hair (7 years ago) (μg/g)	0.41	0.57
HRV		
CVRR%	(1)	0.91
C-CVLF	0.82	0.64
C-CVHF	1.22	0.86
CVF		
Diastolic BP (mmHg)	(1)	0.61
Systolic BP (mmHg)	0.99	0.33
Heart rate (sec^−1^ )	1.06	0.62
BAEP (msec)		
III 20 Hz	1.44	0.93
V 20 Hz	(1)	0.94
III 40 Hz	1.37	0.92
V 40 Hz	0.90	0.90

a The model (see Figure 1) included confounders (age, smoking, alcohol consumption, fish dinners, and BMI), latent mercury exposure, and latent outcomes: HRV (CVRR%, C-CVLF, C-CVHF), CVF (diastolic and systolic BP, and heart rate), and latencies of BAEP (peaks III and V at both 20 Hz and 40 Hz).

**Table 5:** Change in outcome, expressed in percentage of outcome SD (and corresponding 95% confidence intervals) associated with 1 SD increase in exposure.^a^

	Exposure indicator			
Outcome	Blood Hg	Nail Hg	Hair Hg (7 years ago)	Latent Hg
Latent HRV	24.9 (−0.67 to 50.6)*	11.1 (−19.6 to 42.0)	−0.20 (−24.3 to 39.0)	7.80 (−31.7 to 47.3)
CVRR%	43.3 (20.3 to 66.3)**	25.5 (−0.60 to 51.5)*	25.9 (−1.39 to 53.1)*	5.80 (−29.1 to 40.5)
C-CVLF	59.2 (31.2 to 87.3)**	42.7 (11.4 to 74.0)**	39.8 (6.26 to 73.5)**	24.1 (−20.0 to 68.2)
C-CVHF	37.2 (10.7 to 63.9)**	27.6 (−0.29 to 55.5)*	17.2 (−13.1 to 47.5)	9.40 (−22.8 to 41.5)
Latent CVF	23.9 (−12.1 to 60.1)	37.1 (0.46 to 74.3)**	14.5 (−29.3 to 58.0)	38.0 (−5.60 to 81.2)*
Systolic BP	37.5 (5.12 to 69.9)**	23.3 (−10.4 to 56.1)	22.1 (−13.7 to 58.3)	33.8 (−7.16 to 74.6)
Diastolic BP	33.2 (3.15 to 62.9)**	30.9 (0.72 to 61.5)**	14.1 (−18.9 to 46.6)	32.4 (−2.58 to 67.2)*
Heart rate	8.90 (−23.7 to 41.2)	13.3 (−19.4 to 45.7)	17.6 (−16.9 to 52.6)	24.0 (−6.76 to 54.1)
Latent BAEP	−5.9 (−33.9 to 22.1)	13.5 (−18.0 to 45.0)	9.50 (−20.1 to 38.0)	2.4 (−25.2 to 28.8)
III 20 Hz	9.80 (−20.6 to 40.2)	8.20 (−22.8 to 38.2)	26.5 (−5.30 to 58.3)	4.50 (−27.9 to 36.0)
V 20 Hz	17.5 (−11.1 to 46.1)	19.3 (−8.88 to 47.4)	30.3 (23.0 to 59.6)**	11.3 (−21.2 to 43.8)
III 40 Hz	−7.10 (−36.4 to 23.1)	−9.50 (−39.0 to 20.9)	7.70 (−25.2 to 40.6)	−11.6 (−44.5 to 21.3)
V 40 Hz	15.3 (−13.6 to 45.9)	12.3 (−15.4 to 41.6)	32.5 (5.25 to 58.8)**	4.2 (−28.0 to 36.4)
Latent IMT^b^	—	—	—	—
Mean	25.0 (−5.27 to 55.3)	41.9 (14.2 to 69.6)**	33.6 (3.64 to 63.3)**	29.0 (−3.94 to 61.9)*
Maximum	16.8 (−16.0 to 49.7)	34.8 (3.99 to 65.6)**	34.3 (2.50 to 66.0)**	24.7 (−10.5 to 59.8)
Minimum	23.0 (−6.77 to 52.8)	39.0 (11.7 to 66.6)**	29.4 (−0.29 to 59.0)*	24.6 (−7.71 to 56.9)

a All results in the first row of each latent outcome and in the last column of latent Hg exposure were from SEM analyses. All other results were from multiple regression analysis where an individual outcome is regressed on an exposure indicator. Models were adjusted for age, smoking, alcohol consumption, fish dinners, and BMI.

b SEM results not available because of high correlations among the three similar IMT measures.

* *p* < 0.10;

** *p* < 0.05.
